# Traumatic air embolism; a case report and literature review of this rare complication of trauma

**DOI:** 10.1093/jscr/rjae167

**Published:** 2024-03-17

**Authors:** Jonty H Morreau

**Affiliations:** Te Whatu Ora Lakes, Department of General Surgery, PO Box 793, Wellington 6140, New Zealand

**Keywords:** embolism, trauma, penetrating, lung, thorax

## Abstract

Trauma management forms a significant component of any health system. It can affect any body system, and as such knowledge of the wide possible presentations and sequalae are critical. Systemic air embolism (SAE) is a rare presentation in trauma, though it can be associated with significant morbidity and mortality. We present the case of a 23-year-old gentleman with isolated penetrating trauma to the chest who developed widespread neurological insult as a result of SAE, and review historical and proposed contemporary management of SAE.

## Introduction

Trauma is responsible for a significant component of New Zealand Aotearoa’s health presentations. As such, knowledge of the management of trauma and potential subsequent complications is paramount. Embolic disease in trauma is a recognized complication most associated with venous thrombosis and fat emboli syndrome (FES). Intravascular air may in rare circumstances cause an embolic affect with often catastrophic results.

## Case report

A 23-year-old presented to a rural hospital with a single gunshot wound, sustained to his right chest, resulting in a sucking chest wound. He was managed in line with Advanced Trauma Life Support principles and underwent simultaneous assessment and resuscitative interventions, including placement of an intercostal drain in to the right hemithorax and closure of the bullet entry site. Immediate examination and CT scan confirmed no other injury.

In the subsequent hour patient then developed left leg paresthesia and general tonic–clonic seizure activity. An MRI of the brain and spine revealed bilateral superior cerebellar infracts and small left occipital lob infarct. In addition, there was focal cord infarcts seen at C2 and C3 anteriorly and T6 and T7 posterolateral on the left of midline without evidence of locoregional traumatic injury at these sites. The widespread pattern of neurology and imaging was favored to represent embolic insult to the central nervous system. An echocardiogram was performed that did not demonstrate valvular pathology or evidence of cardiac shunt. There was no evidence of deep vein thrombosis or pulmonary embolism. The patient did not demonstrate any vascular risk factors, and subsequent coagulopathy screen was unremarkable.

The patient recovered without active intervention, and following extensive rehabilitation was discharged home with minimal left foot drop as only sequalae of significant neurological insult.

## Discussion

Embolic disease is strongly associated with traumatic pathology. The most common aetiologies are associated with thrombotic disease and FES [1, 2]. Systemic air embolism (SAE) is a recognized, but rare complication of trauma [3]. Venous thrombotic disease rarely occurs within 24 h of traumatic pathology and tends to present as deep vein thrombosis or pulmonary emboli [1]. FES is associated with potentially devastating morbidity or mortality and may affect both the pulmonary and systemic vascular system within 12–36 h of trauma. However, it is almost always associated with fractures or manipulation of long-bones causing the release of fat microglobules which serve as emboli [2].

SAE is a recognized entity in both blunt and penetrating trauma but is more frequent in the latter. It occurs as a result of damage to adjacent bronchial and pulmonary venous structures. It presents most commonly as catastrophic circulatory failure as a result of air emboli within the chambers of the heart and coronary arteries, though isolated neurological sequalae has been reported [4]. Trauma to both airway and pulmonary vasculature structures allow air to enter the vasculature. This will occur at greater rates in patients requiring positive pressure ventilation due to the pressure gradient from airway to vasculature, and these patients can be associated with a rapid and profound deterioration [4]. Injuries close to the hilar regions are at higher risk for SAE because of the proximity of pulmonary vein to large bronchial structures.

SAE is a challenging diagnosis, as there are many competing causes for neurological or circulatory collapse in trauma. Several case reports suggest the identification of air within vascular system is possible on CT, but it is not sensitive enough to exclude the diagnosis [5]. The use of precordial ultrasound has increased the potential for diagnosis of air embolism by visualizing the presence microbubbles within the heart or great vessels, though this requires a skilled sonographer and is best detected via the transoesophageal route [6].

Given the challenging recognition of patients with SAE and the likely rapid cardiovascular deterioration there is little robust evidence supporting appropriate treatment algorithms [4]. Historically immediate thoracotomy and hilar clamping of the affected side has been suggested to remove the source of further air entrapment, but selective lung isolation and differential ventilation may preclude the necessity for thoracotomy [4, 7, 8]. As circulatory collapse may be caused by ventricular outflow obstruction as a result of a large air embolus, placement of the patient in the left lateral decubitus position is another traditional treatment. Animal studies, however, suggest that body positioning does not alter the haemodynamic status of a patient with an air embolus [9].

SAE with neurological consequences is a recognized complication following cardiac or endovascular procedures [5, 10]. Hyperbaric Oxygen Therapy (HBOT) has been utilized to good effect in these situations by constricting pathologic air bubbles thus improving neurological outcomes. This is known as Boyle’s Law tells us that increasing the pressure on a gas bubble in a liquid, the volume of the gas will decrease [11]. Furthermore, by increasing the partial pressure of oxygen in the blood as occurs in HBOT, nitrogen will be encouraged to leave the gas bubble into the adjacent tissues [11]. The positive effects of HBOT are most apparent within therapy is initiated 6 h of SAE though there is evidence of some benefit if initiated within 30 h [3, 12]. Although a recognized sequalae of cardiac or endovascular interventions, the study of HBOT in trauma for SAE is limited to case reports [13].
